# Characterizing the Influence of Confirmation Bias on Web Search Behavior

**DOI:** 10.3389/fpsyg.2021.771948

**Published:** 2021-12-06

**Authors:** Masaki Suzuki, Yusuke Yamamoto

**Affiliations:** Department of Informatics, Shizuoka University, Shizuoka, Japan

**Keywords:** web search, confirmation bias, information behavior analysis, human factor, health information seeking

## Abstract

In this study, we analyzed the relationship between confirmation bias, which causes people to preferentially view information that supports their opinions and beliefs, and web search behavior. In an online user study, we controlled confirmation bias by presenting prior information to participants that manipulated their impressions of health search topics and analyzed their behavioral logs during web search tasks. We found that web search users with poor health literacy and negative prior beliefs about the health search topic did not spend time examining the list of web search results, and these users demonstrated bias in webpage selection. In contrast, web search users with high health literacy and negative prior beliefs about the search topic spent more time examining the list of web search results. In addition, these users attempted to browse webpages that present different opinions. No significant difference in web search behavior was observed between users with positive prior beliefs about the search topic and those with neutral belief.

## 1. Introduction

The credibility of web information has become a serious social issue. For example, Sillence et al. reported that more than half of the health information available on the web has not been verified by experts (Sillence et al., 2004). Therefore, if web search users may believe misinformation, they cannot distinguish correct and incorrect web information.

In addition, problems with web information credibility are amplified due to the personalization of information delivery, e.g., web search engines and recommendation systems. The “filter bubble,” which is phenomenon where users only access information they are interested in due to the optimization of information access, is becoming a social problem because it deprives users of the opportunity to examine information from broader perspectives to facilitate careful and effective decision making (Le et al., 2019; Yamamoto and Yamamoto, 2020).

People can believe incorrect or low-quality information due to “confirmation bias,” which is a concept defined in cognitive psychology. In cognitive psychology, confirmation bias, i.e., the tendency to preferentially view information that is consistent with one's opinions or hypotheses, has a significant impact on decision making (Nickerson, 1998; Kahneman, 2011). Confirmation bias occurs frequently in web searches. For example, assume that user X, who is health conscious, learns on TV that food Y, which uses genetic modification, is harmful to health and distrusts food Y. When user X performs a web search to obtain information about food Y's safety, they unconsciously seek to support the idea that food Y is harmful to their health; therefore, user X will preferentially browse negative information about food Y, even if that information is incorrect or low-quality. Thus, confirmation bias can be a significant problem in web search behavior because confirmation bias that occurs when users search the web for information about food, clothing, housing, and politics can significantly impact society.

There are several studies on the relationship between confirmation bias and web search behaviors (White, 2013; Schweiger et al., 2014; Pothirattanachaikul et al., 2019). For instance, White investigated the impact of prior beliefs on web search behaviors and demonstrated that the prior beliefs of web search users are likely to be strengthened by web search when their prior beliefs about the search topics are not strong (White, 2013). White also found that web search users are more susceptible to positive search results. Pothirattanachaikul et al. studied how opinion polarity and document credibility affect the search behavior and prior belief of web search users (Pothirattanachaikul et al., 2019). They found that web search users spent more time on search tasks when they viewed webpages with opinions that are inconsistent with their existing beliefs. Schweiger et. al. focused on treatment for manic depression and studied the relationship between confirmation bias toward psychotherapy and searchers' belief change on the treatment after reading web pages (Schweiger et al., 2014). Their study suggested that showing experts' evaluation on treatment could reduce confirmation bias and change the prior belief. Like the above studies, many have focused on investigating how confirmation bias influences searcher belief on topics via web searches. However, few studies have characterized the influence of confirmation bias on *behaviors on search engine results pages (SERPs) and webpages* as well as belief change via web searches, based on log-based analysis (e.g., number of clicks, dwell time on webpages, and click depth). Moreover, few studies have examined the relationship between confirmation bias, web search behaviors, and critical information-seeking skills, i.e., information literacy.

In the fields of information retrieval and human-computer interaction, several studies have investigated how to present information to enhance critical information seeking on the web (Liao and Fu, 2014a; Liao et al., 2015; Yamamoto and Yamamoto, 2018, 2020). For instance, Liao et al. revealed that indication of the opinion stance and expertise of the information sender can mitigate the confirmation bias (Liao and Fu, 2014a). Yamamoto et al. proposed the Query Priming system, which facilitates careful information retrieval by showing keywords that evoke critical thinking on web search systems (Yamamoto and Yamamoto, 2018). Query Priming employs keyword auto-completion and keyword suggestion to present search terms that stimulate critical thinking and encourages careful information seeking and decision making. In addition, Yamamoto et al. proposed the Personalization Finder, web browser extension to reveal the effects of web search personalization and promote careful web search practices (Yamamoto and Yamamoto, 2020). The Personalization Finder exposes search results personalized/hidden by web search engines so that searchers can get aware that web search engines provide them with a biased list of web pages according to the searchers' preference. However, these methods were designed for situations where useful meta-information can be obtained to mitigate confirmation bias, e.g., information provider's expertise/perspective, typical search queries used by careful web searchers, and user preference models. If the typical behaviors of web search users with confirmation bias can be identified and compared to those of users with critical information search skills, we believe it will be possible to design web search systems that consider and reduce confirmation bias.

Previously, we conducted a pilot-study to investigate the relationship between confirmation bias and web search behaviors (Suzuki and Yamamoto, 2020). Although the results of that study suggested that people with confirmation bias can perform web search differently to people without the bias, the study design was not sufficiently rigorous to validate the findings because it was difficult to clearly distinguish participants with confirmation bias from those without the bias. Thus, in this study, we quantitatively analyzed the relationship between confirmation bias, information literacy, and web search behavior on health topics by generating pseudo-confirmation bias in participants. We had participants conduct online search tasks by manipulating prior information about health topics to control confirmation. We then analyzed the differences in the web search behaviors of users with and without confirmation bias. We believe it is essential to design information access systems such as web search engines ans web browsers that considers confirmation bias to encourage users to avoid incorrect information for critical health information seeking on the web.

Ennis defined critical thinking as logical and reflective thinking to determine what to believe or do (Ennis, 1987). Ennis also claimed that ideal critical thinkers are disposed to: seek reasons, consider the total situation, look for alternatives, and use logical thinking, e.g., deductive reasoning. Kusumi et al. stated that accurate evaluations of information require searchers to possess critical thinking attitudes and critical thinking skills, e.g., language and reasoning skills (Kusumi et al., 2017). In addition, using the elaboration likelihood model (ELM), Petty et al. indicated that possessing motivation to scrutinize information is a prerequisite for people to utilize critical thinking skills (Petty and Cacioppo, 1986). Confirmation bias can influence people's attitudes about evaluating information. We expect that, if search users have no confirmation bias and do web searches as critical thinkers, to obtain correct and information from the web during web search processes, they will behave in the same manner which the information literacy researchers or librarians think is important. According to Meola (2004) and Yamamoto et al. (2018), the following actions are necessary to obtain correct information on the web: (1) spending more time searching, (2) browsing more webpages for comparison, (3) browse web pages in lower-ranked web search results as well as higher-ranked ones, and (4) checking evidence to support webpage content, such as the expertise of webpage authors, existence of valid references, and the freshness of webpages. Therefore, we set the following hypotheses **H1** and **H2** for our online user study.

**H1** Web searchers with confirmation bias preferentially browse information that is consistent with their beliefs and do not carefully examine which information they should view. Thus, they spend less time browsing the search results list and preferentially browse higher-ranked pages in the results.
**H2** Web searchers with confirmation bias only view information that is consistent with their beliefs and do not browse information carefully. Thus, they spend less time browsing webpages and view fewer webpages.

As mentioned above, the ELM theory indicates that if people are more willing to understand information about a topic, they often make more efforts to scrutinize its quality and modify their prior belief if necessary (Petty and Cacioppo, 1986). On the other hand, White found that web search users often strengthen their own beliefs through search (White, 2013). Based on these two studies, we also set the following hypothesis **H3** for the user study.

**H3** Web searchers with confirmation bias do not change their beliefs significantly when they search the web, compared to users without confirmation bias.

Lopes et al. analyzed the relationship between health literacy and web search behavior using eye-tracking analysis (Teixeira Lopes and Ramos, 2020). They found that web search users with higher health literacy visited more webpages and spent more time reading webpages. Furthremore, Yamamoto et al. revealed that the higher health information literacy web searchers have, the more tolerant they are for cognitive biases in web searches (Yamamoto et al., 2018). Therefore, we set the following hypothesis **H4**.

**H4** The degrees of **H1**, **H2**, and **H3** are influenced by the web search user's degree of information literacy.

## 2. Materials and Methods

This section describes the methodology employed to analyze the impact of confirmation bias and information literacy on web search behavior. The details of the experiment are described in the following. Note that we refer to the group with negative beliefs about the search topic as the biased(−) group, we refer to the group with positive beliefs as the biased(+) group, and we refer to the group with no bias as the neutral group.

### 2.1. Procedures

We conducted an online user study in Japanese according to the following procedure: (1) user registration; (2) prior belief questionnaire; (3) presentation of prior information about the search topic; (4) search task; and (5) post-task questionnaire.

First, the participants visited the experimental site prepared by our laboratory after they registered as users at Lancers.jp, which is a Japanese crowdsourcing service^1^. Then, the participants answered a questionnaire on their prior beliefs about a given search topic. In the prior belief questionnaire, we asked the participants to answer the following question on a five-point Likert scale: “How do you feel about the safety of eating GM (genetically modified) foods?” (“1. Danger;” “2. Somewhat danger;”, “3. Neither danger nor safe;” “4. Somewhat safe;” to “5. Safe”).

We then assigned participants to specific experimental conditions based on their answers regarding their prior beliefs about the search topic.

- biased(−) group: Participants who answered “Dangerous” or “Somewhat dangerous.”
- biased(+) group: Participants who answered “Safe” or “Somewhat safe.”
- neutral group: Participants who answered “Neither danger nor safe.”

Next, we presented prior information to strengthen the participants' prior beliefs to introduce confirmation bias during the search task. Here, the presented information comprised a section 1 that described the search task and a section about GM foods. Note that we used the same description for all participants; however, we presented different descriptions about GM foods depending on the participants' prior beliefs.

The introduction for the search task is as follows.

*You pick up a bottle of rapeseed oil that was on sale, and you notice a label that states that “it may contain GM rapeseed.” You have always been a little curious about GM foods. Then, you asked your friend to give you some advice about GM foods*.

In addition, we presented different information to strengthen the participants' prior beliefs depending on the experimental group. The information presented to each group is described as follows.

- biased(−) group: This group was shown a 200-word negative description of GM foods (e.g., “Europe has strict regulations against GM foods.”) and a 2-min video^2^ against GM foods.
- biased(+) group: This group was shown a 200-word positive description of GM foods (e.g., “Japan's Ministry of Health, Labor and Welfare (MHLW) carries out strict screening, and many Japanese people eat GM foods.”) and a 2-min video^3^ supporting GM foods.
- neutral group: This group was shown the negative and positive information presented to the biased(−) and (+) groups so that the participants in this group could understand there is controversy about whether or not GM foods are safe to eat.

To ensure all participants viewed the preliminary information, we asked them to summarize the content in approximately 100 words after viewing the video.

The participants performed the search task after viewing the preliminary information. The following instructions were presented to the participants when they began the search task.

*Follow the steps below to complete the task of investigating whether or not it is safe to eat GM foods. Click on the “Start the search” button below and browse a list of search results and their links. When you have reached a satisfactory conclusion about “whether it is safe to eat GM foods,” please stop searching the web and report your final opinion and the reasons for it in the form*.

After participants clicked the “Start the search” button, they browsed a search engine results page (SERP) and the documents linked from the SERP to collect information about the safety of eating GM foods.

When the participants were satisfied with the obtained information, they completed the search and reported their responses to the search task (posterior beliefs). Here, the participants were asked to answer a questionnaire about whether it is safe to eat GM foods using the same five-point Likert scale used in the prior belief questionnaire. Note that we did not set a time limit in this search task because the goal was to analyze how participants searched and browsed at their discretion.

After completing the search task, the participants answered the post-task questionnaire about health literacy and demographic characteristics. We used the eHealth Literacy Scale (eHEALS) to survey information literacy on health topics, i.e., the ability to search for reliable health information on the web (health literacy) (Norman and Skinner, 2006). The participants answered the eight questions on a five-point Likert scale (“1: I never agree” to “5: Completely agree”). Here, we used the total eHEALS score as an indicator of the degree of each participant's health literacy. In addition, in the demographic characteristics questionnaire, we investigated the participants' gender, age, and educational background.

### 2.2. Search Task and Search Results List

We set a search task for a search topic that increases the polarity's variance and degree of prior beliefs. In this experiment, we selected “GM foods,” which is a controversial topic in Japan, as the search topic.

**Figure 1:**
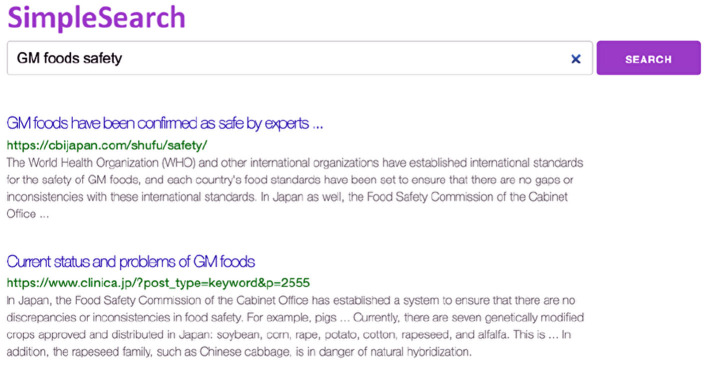
SERP presented to participants in the user experiment.

In the search task, we presented the participants with a list of search results that imitated those returned by common web search engines, e.g., Google^4^ and Yahoo!^5^ The search result list included 30 search results prepared in advance for the given search topic. Figure 1 shows the search result list used in the search task.

**Figure 2:**
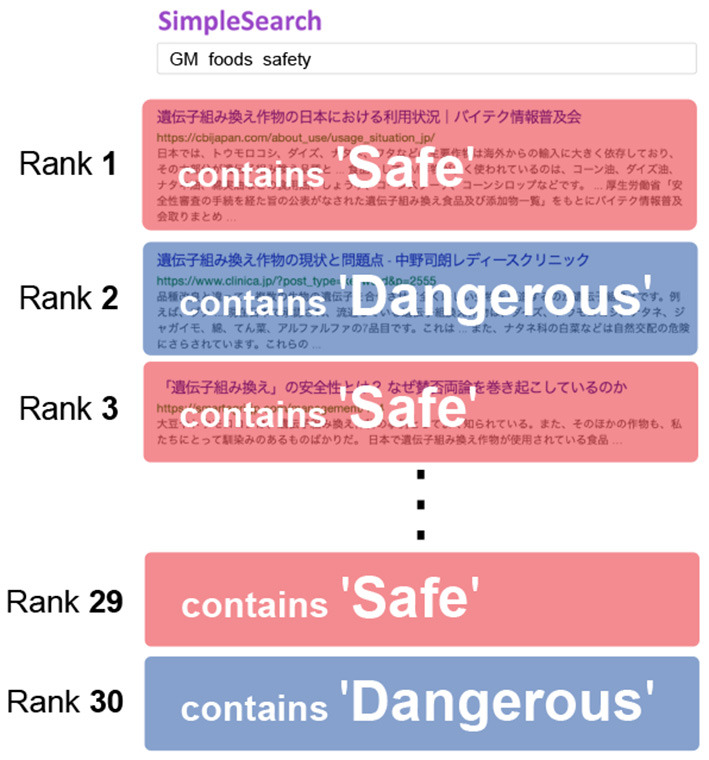
Allocation of search results on SERP. Red and blue search results contain the terms “safe” and “dangerous” in their title or summary, respectively.

Before starting the task, we performed a Google search using the queries “GM foods safe” and “GM foods dangerous” to obtain 15 search results containing the words “safe” and “dangerous” in the title or summary (referred to as a snippet). We defined the search results collected by the former query as *search results containing positive information about prior beliefs* and search results collected by the latter query as *search results containing negative information about prior beliefs*. We then created a list of search results by alternately displaying the results of the two queries from the top (Figure 2). We displayed the positive and negative results alternately to present both types of information as equally as possible to the participants. Although the search results imitate the results screen of a general web search, the system was configured such that participants could not modify the search queries.

When the participants clicked each search result, an archived version of the corresponding webpage was displayed. Here, we embedded JavaScript code in the archived webpages to measure the browsing time on each webpage. In addition, we disabled hyperlinks in the documents; thus, the participants could not view documents other than those displayed in the search results list. As a result, we measured the page browsing time for only the webpages in the search result list.

### 2.3. Participants

We recruited 300 Japanese participants using Lancers.jp. We excluded data for participants who failed to complete the task or worked on the task multiple times for some reasons. After selecting the data to exclude, we used the data from a total of 275 participants in our analysis.

We then assigned the participants to specific groups according to their prior beliefs. In the biased(−) group, 148 participants completed the task, and 96 and 31 participants completed the task in the neutral group and biased(+) group, respectively. Note that we paid 100 Japanese yen to each participant who completed the task.

### 2.4. Monitored Data

We collected data on the following items during the search task to analyze the relationship between confirmation bias and web search behavior.

- Dwell time on search engine results page (SERP)
- Dwell time on webpages
- Search session time
- Clickthrough of search results.

The dwell time on SERP is the total time the participants browsed the SERP, and the dwell time on webpages is the time the participants spent browsing the webpages linked from the SERP. The search session time is the total time the participants browsed the webpages and SERP, and the clickthrough of search results is the information in the search results the participants clicked on the SERP. The clickthrough information includes the title, summary text, URL, search result rank, and belief polarity (i.e., whether the search result contains “safe” or “dangerous” in the title or summary text). We set up these indicators in reference to the paper by White et al., which analyzed web search behavior logs (White and Morris, 2007).

### 2.5. Analyses

We employed the generalized linear mixed model (GLMM) (Barr et al., 2013) to analyze the users' behavioral logs. The GLMM can separate the main effect of the intervention from the random effect, which is the effect of individual differences among the participants and tasks. Note that the GLMM can analyze small-scale data more accurately than methods that employ frequentist statistics (Kay et al., 2016). The GLMM is becoming an increasingly established method to model user behavior in the information retrieval and human-computer interaction fields (Kim et al., 2017). In this study, we modeled the behavioral data using the GLMM extended by the Bayesian statistical model.

Here, we assumed that search session time and dwell time on SERP follow a Weibull distribution (Liu et al., 2010). We also assumed that the number of page views and maximum click depth follow a Poisson distribution, and that the amount of belief change follows a normal distribution.

In the GLMM, we set the two factors, i.e., confirmation bias (condition) and health literacy score (eHEALS), as the main effects and the participant as a random effect. Following the literature (Barr et al., 2013), we modeled the behavioral indicator measured in the user experiment as follows^6^:


$$\begin{array}{l} \text{Y}\sim \text{Cond}+\text{eHEALS}+\text{Cond}:\text{eHEALS}\\ \;+\left(1+\text{Cond}+\text{eHEALS}+\text{Cond}:\text{eHEALS}|\text{Participant}\right), \end{array}$$

where Y is the target variable, Cond is a binary value indicating the presence or absence of confirmation bias for each participant, and eHEALS is the health literacy score. Here, (x|y) means that y is a random effect of x.

We used the highest density interval (HDI) as a measure to investigate the effect of the condition and eHEALS factors. The HDI represents the possible range of the parameter, where the parameter is considered effective if the HDI does not contain zero. Note that this is equivalent to rejecting the null hypothesis in frequentist statistics. Following Kruschke's point, we set the HDI for the parameter to be effective at 90% (Kruschke, 2014).

We used a non-parametric test to analyze the results of the post-task questionnaire.

## 3. Results

From the user experiment, we collected behavioral and questionnaire data from the 275 participants. Here, we describe the results of the analyses of the behavioral data, the pre-task questionnaire, and post-task questionnaires.

We analyzed the effects of two factors, i.e., the presence of condition and eHEALS, on search/browsing behavior and information scrutiny perspectives. Here, we set three levels for the condition: (1) with negative confirmation bias (biased(−) group), (2) without confirmation bias (neutral group), and (3) with positive confirmation bias (biased(+) group). We then analyzed the differences between the biased(−) and biased(+) groups compared to the neutral group.

Table 1 shows the mean values and standard deviations of the various behavioral indices for each condition.

**Table 1:** Mean and standard deviation of condition in each behavioral index.

	**Condition**		
**Behavioral index**	**BIASED(−)**	**NEUTRAL**	**BIASED(+)**
Search session time (second)	446.6 (446.8)	437.0 (379.3)	269.7 (314.0)
Dwell time on SERP (second)	73.0 (86.6)	75.7 (63.4)	58.4 (82.9)
Maximum dwell time on webpage (second)	146.4 (118.5)	155.5 (144.9)	93.6 (58.5)
Maximum click depth	11.2 (9.2)	12.5 (9.9)	8.9 (9.4)
Number of page views	5.0 (4.7)	5.3 (5.2)	5.1 (7.5)
Number of page views(−)	2.8 (2.6)	2.9 (2.8)	4.2 (5.0)
Number of page views(+)	3.0 (2.4)	3.2 (2.6)	3.1 (3.6)
Belief change	0.39 (1.15)	0.26 (0.99)	−0.35 (1.02)

### 3.1. Search Session Time

To analyze how carefully participants performed their search and browsing behavior, we compared the search session time for each group of participants. Table 2 shows that the 90% HDI of the coefficient of the condition did not contain zero in the analysis comparing the biased(−) and neutral groups. Note that this is equivalent to rejecting the null hypothesis in frequentist statistics.

**Table 2:** GLMM results compared to neutral group.

	**BIASED(−)**			**BIASED(+)**		
**Behavioral Index**	**Condition**	**eHEALS**	**Interaction**	**Condition**	**eHEALS**	**Interaction**
Search session time	**-1.01** **[-1.96, -0.14]**	-0.02 [-0.05, 0.01]	0.01 [ -0.01, 0.04]	0.28 [-1.36, 1.91]	-0.02 [-0.05, 0.01]	-0.04 [-0.10, 0.03]
Dwell time on SERP	**-1.00** **[-1.82, -0.11]**	4.58*e*^−3^ [-0.02, 0.03]	**0.05** **[ 0.01, 0.08]**	0.89 [-0.57, 2.35]	4.58*e*^−3^ [-0.02, 0.03]	-0.06 [-0.12, 0.02]
Maximum dwell time on page	-0.54 [-1.37, 0.32]	-0.02 [-0.04, 0.01]	0.02 [-0.01, 0.05]	0.40 [-0.92, 1.78]	-0.02 [-0.04, 0.01]	-0.04 [-0.09, 0.02]
Maximum click depth	**-1.02** **[-1.97, -0.15]**	1.75*e*^−3^ [-0.03, 0.03]	**0.04** **[ 0.00, 0.07]**	-0.20 [-1.76, 1.34]	1.75*e*^−3^ [-0.03, 0.03]	-0.01 [-0.07, 0.05]
Number of page views	-0.45 [-1.29, 0.37]	9.92*e*^−4^ [-0.02, 0.03]	0.01 [-0.09, 0.04]	0.24 [-1.43, 1.79]	9.92*e*^−4^ [-0.02, 0.03]	-0.02 [-0.09, 0.04]
Number of page views(−)	-0.14 [-1.15, 0.82]	8.52*e*^−3^ [-0.02, 0.04]	5.33*e*^−3^ [-0.03, 0.05]	-0.23 [-3.12, 2.94]	8.52*e*^−3^ [-0.02, 0.04]	0.01 [-0.12, 0.14]
Number of page views(+)	**-0.84** **[-1.60, -0.09]**	-9.99*e*^−3^ [-0.03, 0.01]	**0.03** **[ 0.00, 0.06]**	-0.12 [-1.55, 1.25]	-9.99*e*^−3^ [-0.03, 0.01]	4.58*e*^−3^ [-0.06, 0.05]
Belief change	0.50 [-0.55, 1.44]	9.03*e*^−3^ [-0.02, 0.04]	-0.34 [-1.80, 1.20]	0.39 [-2.12, 2.65]	9.03*e*^−3^ [-0.02, 0.04]	-0.01 [-0.07, 0.05]

*Numbers represent the median and interval of 90% HDI. Bold numbers do not contain zero in the 90% HDI*.

These results demonstrate that the biased(−) group tended to have shorter search session time than that of the neutral group. However, the 90% HDI of the coefficients of the eHEALS and interaction contained zeros, which is equivalent to not rejecting the null hypothesis in frequentist statistics. In addition, we observed that eHEALS and interaction had no effect on the search session time.

The 90% HDI for condition, eHEALS, and interaction coefficients contained zero in the analysis comparing the biased(+) and neutral groups. Therefore, the presence or absence of positive confirmation bias had no effect on the search session time.

### 3.2. Dwell Time on SERP

We compared the SERP browsing time to analyze how carefully the participants browsed the list of search results while collecting information. We found that the 90% HDI of the coefficient of the condition and interaction did not contain zero in the analysis comparing the biased(−) and neutral groups.

**Figure 3:**
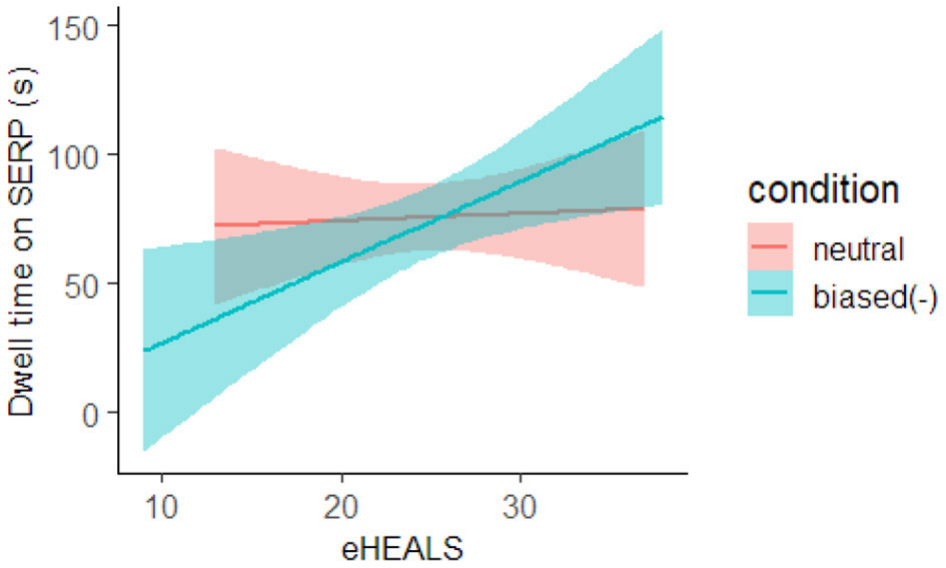
Estimated effect of condition and eHEALS on SERP dwell time. The red line represents the neutral group, and the blue line represents the biased(−) group. The background color indicates the confidence interval.

The interaction was confirmed; thus, we conducted a simple main effect analysis, and the results are shown in Figure 3. As can be seen, when the participant's eHEALS was low, the biased(−) group tended to spend less time browsing SERP compared to the neutral group. However, when the eHEALS was high, the biased(−) group tended to spend more time browsing the SERP compared to the neutral group.

As shown in Table 2, the 90% HDI of the coefficients of condition and interaction contained zero in the analysis comparing the biased(−) and neutral groups. Therefore, the presence or absence of positive confirmation bias had no effect on SERP dwell time.

### 3.3. Maximum Dwell Time on Webpage

To analyze how carefully the participants browsed the webpages in the SERP, we compared the participants' maximum webpage browsing time during the search task. Compared to the neutral group, the 90% HDI of the condition, eHEALS, and interaction coefficients contained zero for the biased(−) and biased(+) groups (Table 2), which indicates that the presence or absence of confirmation bias had no effect on maximum dwell time.

### 3.4. Number of Page Views

We also evaluated the number of webpages viewed by the participants during the search task to analyze how intensively the participants attempted to collect evidence when they assessed the truth of the given search topic. Compared to the neutral group, the 90% HDI of the condition, eHEALS, and interaction coefficients contained zero for both the biased(−) and biased(+) groups (Table 2), which indicates that the presence or absence of confirmation bias had no effect on the number of page views.

We also analyzed the extent to which participants viewed webpages containing information that was consistent with their prior beliefs. Here, the number of clicks on a webpage that included the word “dangerous” in the title or summary of the search result was defined as the number of pageviews(−). In addition, we defined the number of clicks on a webpage that included the word “safe” as the number of pageviews(+).

For the number of pageviews(−), the 90% HDI of the condition, eHEALS, and interaction coefficients contained zero for both the biased(−) and biased(+) groups (Table 2), which indicates that the number of pageviews(−) was not affected by the presence or absence of confirmation bias.

**Figure 4:**
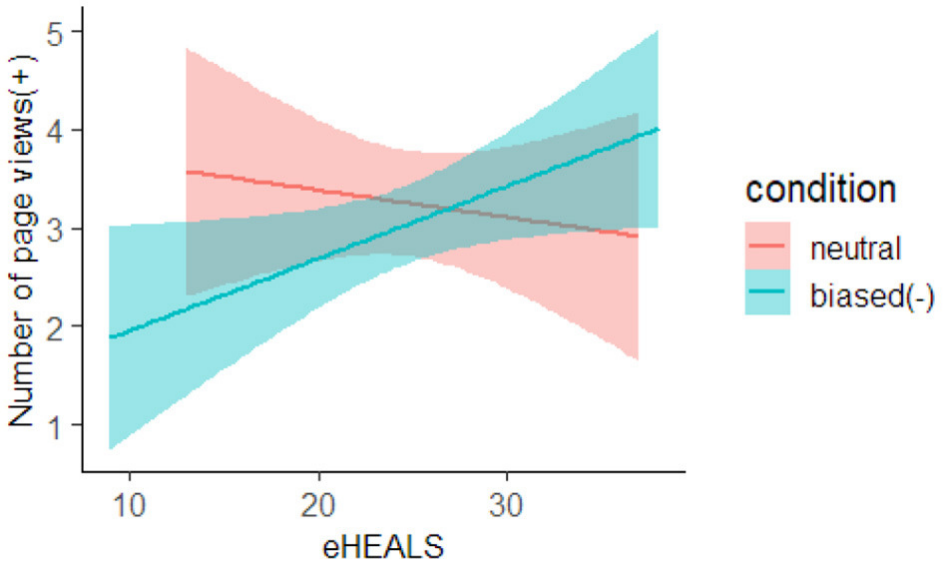
Estimated effect of condition and eHEALS on number of page views(+). The red line represents the neutral group and the blue line represents the biased(−) group. The background color indicates the confidence interval.

For the number of pageviews(+), the 90% HDI of the condition and interaction coefficients did not contain zero in the analysis comparing the biased(−) and neutral groups (Table 2). Here, as we observed the interaction, we conducted a simple main effect analysis, and the results are shown in Figure 4. As can be seen, when the participant's eHEALS was low, the biased(−) group tended to have fewer pageviews(+) than the neutral group. However, when the participant's eHEALS was high, the biased(−) group tended to have more pageviews(+) than the neutral group.

For the number of pageviews(+), the 90% HDI of the condition and interaction coefficients did not contain zero in the analysis comparing the biased(+) and neutral groups (Table 2). This indicates that the presence or absence of positive confirmation bias had no effect on the number of pageviews(+).

### 3.5. Maximum Click Depth

**Figure 5:**
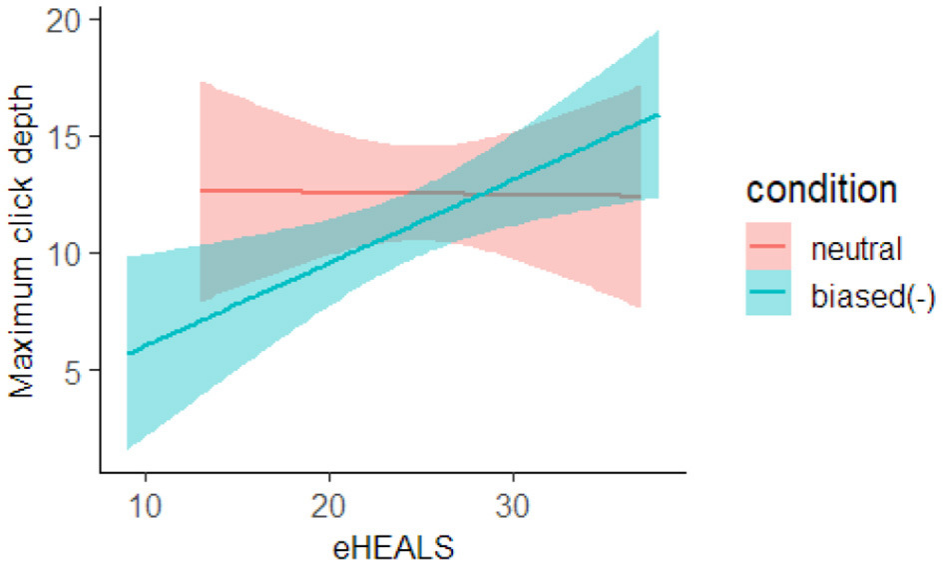
Estimated effect of condition and eHEALS on maximum click depth. The red line represents the neutral group, and the blue line represents the biased(−) group. The background color indicates the confidence interval.

To analyze how deeply the participants scanned the search result list, we investigated the order of the search results the participants clicked on to analyze the maximum search result rank, i.e., the maximum click depth. Table 2 shows that the 90% HDI of the condition and interaction coefficients did not contain zero in the analysis comparing the biased(−) and neutral groups. Here, we conducted a simple main effect analysis because we observed the interaction, and the results are shown in Figure 5. As can be seen, when the participant's eHEALS was low, the biased(−) group tended to click on higher search results than the neutral group. However, when the participant's eHEALS was high, the biased(−) group tended to click on lower search results than the neutral group.

As shown in Table 2, the 90% HDI of the condition, eHEALS, and interaction coefficients contained zero in the analysis comparing the biased(+) and neutral groups, which indicates that the presence or absence of positive confirmation bias had no effect on the maximum click depth.

### 3.6. Belief Change

We evaluated the difference between the posterior and prior beliefs to analyze the extent to which the participants' prior beliefs changed as a result of the search task. Table 2 shows that the 90% HDI of the condition, eHEALS, and interaction coefficients included zero for both the biased(−) and biased(−) groups compared to the neutral group. These results indicate that participants did not change their prior beliefs much over the course of the search task regardless of the presence of positive or negative confirmation bias.

## 4. Discussion

### 4.1. Hypothesis Verification

In this study, we analyzed the SERP browsing time and maximum click depth to verify **H1** regarding the webpage selection behavior. The results demonstrated that when the participant's eHEALS score was low, the biased(−) group spent less time browsing the SERPs than the neutral group, tended to click on the higher (shallower)-ranked search results, and viewed pages that were inconsistent with their prior belief less frequently. When the participants' eHEALS score was high, the biased(−) group spent more time browsing the SERPs than the neutral group, tended to click on lower (deeper)-rank search results, and viewed pages that were inconsistent with their prior belief more often. In contrast, no difference was observed in SERP browsing time and maximum click depth for the biased(+) and neutral groups.

The eHEALS score is a scale that reflects the information literacy required to obtain and view health information on the web carefully (health literacy). Therefore, even if participants with high health literacy had negative confirmation bias for the search topic, they could reduce the negative confirmation bias and carefully select webpages to view. In contrast, when participants with low health literacy had negative confirmation bias about the search topic, they could not reduce the negative confirmation bias and spent much attention and time selecting the webpages to view from the search result list. Thus, we believe that hypotheses H1 and H4 regarding webpage selection are supported only when web search users have negative confirmation bias for the given search topic.

We also analyzed the maximum page browsing time and number of page views to verify **H2** regarding webpage browsing behavior. Here, we did not find that maximum page browsing time was affected by confirmation bias. For the number of page views, the biased(−) group with low eHEALS score tended to view fewer webpages containing the word “safe” in the title or summary text compared to the neutral group. In contrast, the biased(−) group with a high eHEALS score tended to view more webpages with the word “safe” in the title or summary text compared to the neutral group. Similar to the results of the analyses of dwell time on SERP and maximum click depth, these results suggest that the participants with low health literacy could not control the effects of confirmation bias when they had negative confirmation bias for the given search topic. In addition, the results indicate that the participants did not actively browse webpages that were inconsistent with their belief (i.e., webpages that refers to GM foods as safe). In contrast, participants with high health literacy were able to reduce the impact of negative confirmation bias and actively browsed webpages that were inconsistent with their prior belief in the search results. Therefore, we believe that H2 and H4 were supported only when the participants had a negative confirmation bias about the given search topic.

We analyzed the difference in belief before and after performing the web search task to verify **H3** regarding belief change after web searches. The results demonstrate that no significant difference was observed in terms of the amount of belief change in the biased(−) and biased(+) groups compared to the neutral group. Thus, we consider that **H3** was not supported. The results for **H1** and **H2** indicate that even if web search users with high health literacy had negative confirmation bias for the given search topic, they viewed pages with different positions actively. Thus, the results for **H3** suggest that it is difficult for users with high health literacy to change their beliefs in a significant way, even if they are able to reduce the negative effects of confirmation bias and perform careful search browsing behavior.

Finally, we discuss the differences in the various behavioral indexes only for the biased(−) group. Rozin et al. found that humans are more influenced by negative information than positive information (Rozin and Royzman, 2001); therefore, we expected that the negative confirmation bias for search topics would impact search browsing behavior more than positive confirmation bias. We found that the biased(−) group was more affected by confirmation bias than the biased(+) group, and the values of the various behavioral indexes decreased significantly compared to those of the neutral group.

In summary, our study revealed that when web searchers with poor health literacy have negative prior beliefs about health topics, they could not examine web search results and preferentially view web pages supporting their beliefs. On the other hand, if web searchers with high health literacy have negative prior beliefs about health topics, they could spend more time examining web search results and browsing web pages that present different opinions. However, the study results indicate that their prior belief could not change so much even if they browse various opinions. In the case where web searchers have positive prior beliefs about health search topics, we did not observe the relationship between health literacy and web search behaviors.

The study results imply several points to design classes and information access systems for critical information seeking on the web. Firstly, we might need to develop educational classes related to information literacy so that people can reflect and improve their web search behaviors toward critical information seeking. It might be good to collaborate with computer scientists to develop a function on web search/browsing systems that general web searchers can use to reflect their search behaviors. As our study revealed, web searchers with poor health literacy did not often examine web search results and compare them with various web pages. Consequently, they lost opportunities to check if their prior belief could be wrong or disputed. Bateman et al. proposed a search user interface that summarizes web search histories of users and revealed that the interface could help users modify their search behavior to improve search performance (Bateman et al., 2012). For supporting web searchers with low health literacy, one possible application is a web browser extension to visualize user behavior tendencies in order to encourage people to improve deficiencies relative to behaviors of web searchers with high health literacy.

The second point is prediction of the extent of health literacy. Our study revealed that if web searchers with poor health literacy have negative prior beliefs about health search topics, they often make less effort to examine web search results than those with high health literacy. For supporting web searchers with poor health literacy efficiently, we need a method to find such searchers. We observed specific web search behaviors to distinguish web searchers with poor health literacy and those with high literacy (e.g., dwell time on SERP, number of page views, and maximum click depth) through the online study. In the field of computer science, machine learning is a popular technique to make predictions with data. We plan to apply machine learning techniques to web search behavior data to build a predictor for the health literacy of web searchers.

The third point is mitigation of confirmation bias in web searches. Although our study suggests that it is difficult for web searchers to change their prior beliefs, we need to support web searchers mitigating their confirmation bias or doing web searches objectively. One possible application is interactive chat-bot systems that ask web searchers which evidence supports the belief and show contradictory opinions while searching for web information. If computer scientists collaborate with experts from the field of health psychology, we believe that they can develop such systems and contribute to reducing confirmation bias.

### 4.2. Limitations

To realize more accurate analyses, at least two issues must be considered and improved in this user experiment. The first is the generalizability of the results of the online study. In this study, we considered “GM foods” as a search topic in the health field. To confirm whether this study's findings can be generalized to other topics, we must conduct search task experiments in other fields and examine the effects of confirmation bias in each field.

The second issue is the quality of the webpages in the list of search results in the given search task. In our user experiment, we used the results of a Google search with a query pair of the words “safe” or “dangerous” and “GM foods” to create the list of search results. However, when we investigated the domains of the collected webpages, we found that many of the webpages containing the word “safe” were authorized by public organizations, which are generally considered reliable. The “GM foods” chosen as the search topic in this user experiment represents foods that have been confirmed as safe by the Ministry of Health, Labor, and Welfare in Japan (MHLW). Therefore, the list of results including the word “safe” collected by the Google search also contained a significant amount of information from national public organizations, e.g., the MHLW. According to Liao et al., even if information is inconsistent with one's beliefs, users are more likely to view the information if the information provider is identified as having a high level of expertise (Liao and Fu, 2014b). In other words, users with negative confirmation bias may be more likely to click on positive information if it contains reliable information regardless of the polarity of their beliefs. Therefore, it is difficult to precisely analyze why participants with negative confirmation bias viewed the search results containing the word “safe” actively in the current experimental design. Thus, we must conduct user experiments by creating search results for both negative and positive information with the same level of reliability.

## 5. Conclusion

In this paper, we have described an online experiment using crowdsourcing that was conducted to identify web search behaviors in consideration of confirmation bias. To divide users into groups with and without confirmation bias, we provided the participants with prior information to manipulate their impressions of the given search topic. We then analyzed the logs of their search and browsing.

We found that participants with negative beliefs about the given search topic often spent less time browsing the search result list page, clicked on higher-ranked search results, and did not browse search results about positive opinions when they had low health literacy. In contrast, participants with high health literacy, even if they had negative beliefs about the given search topic, often spent more time browsing the search results page, scanned lower ranked search results, and browsed more actively for search results containing positive opinions. However, the results also suggest that it was difficult for participants with high health literacy to remove the negative effects of confirmation bias and change their beliefs, even if they were able to perform careful search browsing behavior. We conclude from these results that web searchers with confirmation bias are unlikely to change their prior beliefs even if they spend a lot of effort searching for information. Therefore, we consider that the most important issue is to design a function on web access systems that supports web searchers to mitigate confirmation bias. Moreover, we need to develop a function of the systems to detect web searchers with poor health literacy and improve their health literacy and web search behaviors toward critical information seeking on the web.

In the future, we plan to challenge the following several issues based on our study results. First, we must conduct additional user experiments with different search topics and search result lists to obtain a deeper understanding of user web search behaviors in consideration of confirmation bias and generalize our findings to other fields. Secondly, we need to develop a function on web search/browsing systems that general web searchers can use to reflect their search behaviors toward critical information seeking. Furthermore, we need to build a system that predicts the health literacy of web searchers and encourages the searchers with poor health literacy to make more efforts for critical web searches. Finally, we need to support web searchers mitigating their confirmation bias by showing contradictory opinions in web searches.

## Data Availability Statement

The raw data supporting the conclusions of this article will be made available by the authors, without undue reservation.

## Ethics Statement

Ethical review and approval was not required for the study on human participants in accordance with the local legislation and institutional requirements. Written informed consent for participation was not required for this study in accordance with the national legislation and the institutional requirements.

## Author Contributions

MS and YY contributed to conception and design of the study. MS developed an experimental system and wrote the first draft of the manuscript. YY performed the statistical analysis. Both authors contributed to manuscript revision, read, and approved the submitted version.

## Funding

This work was supported by JSPS KAKENHI Grant Numbers JP18H03244, JP21H03554, and JP21H03775.

## Conflict of Interest

The authors declare that the research was conducted in the absence of any commercial or financial relationships that could be construed as a potential conflict of interest.

## Publisher's Note

All claims expressed in this article are solely those of the authors and do not necessarily represent those of their affiliated organizations, or those of the publisher, the editors and the reviewers. Any product that may be evaluated in this article, or claim that may be made by its manufacturer, is not guaranteed or endorsed by the publisher.

## Footnotes

^1^https://www.lancers.jp/.

^2^https://www.youtube.com/watch?v=umXN64zIH-8 (in Japanese).

^3^https://www.youtube.com/watch?v=zMnX3qS6Dj4 (in Japanese).

^4^https://www.google.co.jp/.

^5^https://www.yahoo.co.jp/.

^6^The brms package in R was used for modeling.
